# Rapid Identification of High-Temperature Responsive Genes Using Large-Scale Yeast Functional Screening System in Potato

**DOI:** 10.3390/plants12213712

**Published:** 2023-10-28

**Authors:** Ke Wang, Shiqi Wen, Lina Shang, Yang Li, Ziyan Li, Weixi Chen, Yong Li, Hongju Jian, Dianqiu Lyu

**Affiliations:** 1 Integrative Science Center of Germplasm Creation in Western China (CHONGQING) Science City, Southwest University, Chongqing 400715, China; 2 College of Agronomy and Biotechnology, Southwest University, Chongqing 400715, China; 3 Agriculture College, Anshun University, Anshun 561000, China; 4 State Cultivation Base of Crop Stress Biology for Southern Mountainous Land, Southwest University, Chongqing 400715, China; 5 Chongqing Key Laboratory of Biology and Genetic Breeding for Tuber and Root Crops, Chongqing 400715, China

**Keywords:** potato, high-temperature stress, yeast, gene screening, large scale yeast functional screening system

## Abstract

As the third largest global food crop, potato plays an important role in ensuring food security. However, it is particularly sensitive to high temperatures, which seriously inhibits its growth and development, thereby reducing yield and quality and severely limiting its planting area. Therefore, rapid, and high-throughput screening for high-temperature response genes is highly significant for analyzing potato high-temperature tolerance molecular mechanisms and cultivating new high-temperature-tolerant potato varieties. We screened genes that respond to high temperature by constructing a potato cDNA yeast library. After high-temperature treatment at 39 °C, the yeast library was subjected to high-throughput sequencing, and a total of 1931 heat resistance candidate genes were screened. Through GO and KEGG analysis, we found they were mainly enriched in “photosynthesis” and “response to stimuli” pathways. Subsequently, 12 randomly selected genes were validated under high temperature, drought, and salt stress using qRT-PCR. All genes were responsive to high temperature, and most were also induced by drought and salt stress. Among them, five genes ectopically expressed in yeast enhance yeast’s tolerance to high temperatures. We provide numerous candidate genes for potato response to high temperature stress, laying the foundation for subsequent analysis of the molecular mechanism of potato response to high temperature.

## 1. Introduction

By 2050, food demand will increase significantly according to the Food and Agriculture Organization. Crop productivity usually achieves less than half of its maximum potential as it is significantly affected in natural and agricultural systems by multiple stresses such as salinity, drought, extreme temperatures (freezing and heat), mineral deficiency, and toxicity [1]. Such adverse environmental factors are likely to intensify in coming decades because of global warming and climate changes. Therefore, to ensure global food security, we must urgently screen functional genes that respond to stress and then use them to cultivate stress-resistant crops. Among these stresses, high temperature is a continuing threat to crop yields and food security and requires urgent attention.

As the world’s third most important non-grain food crop, potatoes are the staple food crop for most developing countries (http://faostat.fao.org/ (accessed on 23 March 2023)) and cultivated from cool-temperate lowlands to mid elevations of tropical regions [2]. Potato tubers are an ideal food crop because they are rich in starch, protein, and vitamin C and low in fat [3]. Additionally, potatoes are widely used in the food industry (e.g., as fries and chips) and are used as industrial raw materials [4]. However, potato production is predicted to decrease by 9–18% in most parts of the world due to the diverse abiotic stresses, especially high temperatures [5]. High temperature significantly affects potato growth and development (the ability of seed tubers to sprout), tuberization (limited tuber induction and development) and potato quality (reduced carbon portioning to growing tubers) [5]. Furthermore, prolonged high-temperature stress can lead to potato deformities [6]. Therefore, it is very important to screen high-temperature-responsive genes and analyze molecular mechanisms for breeding high-temperature-tolerant potatoes.

There is copious literature related to various mechanisms involved in high-temperature stress in various plants, especially in model plants such as *Arabidopsis* and *Oryza sativa* [7,8]. Over the last few decades, numerous key functional factors, such as transcription factor, heat shock factors, heat shock proteins, phosphatases, and kinases have been characterized and summarized in various reviews [9]. To screen and isolate candidate genes, bioinformatics, map-based cloning, cloning by homology, transcriptomics analysis, and biochemical methods have been frequently used in diverse plant research studies [10,11,12]. Stress response is conservative in eukaryotes, but in yeast it is much simpler to screen stress-responsive genes, and so expressing plants’ cDNA in the yeast system has been applied in recent years. Heterologous expression of exogenous genes in yeast is tested to determine whether they can enhance the yeast’s tolerance to stress. High-throughput screening of resistance-related genes within the genome was conducted through resistance gradient experiments combined with next-generation sequencing technology [13]. *BveIF1A*, screened from a sugar beet cDNA library, was functionally characterized to increase salt tolerance in yeast and plants. An cDNA library was constructed from salt-stressed *Jatrpha curcas* to screen salt tolerance genes, and 32 were identified as candidates [13,14]. In potato, two libraries were constructed to screen drought-responsive and high-temperature-responsive genes, and 69 drought-responsive and 95 heat-responsive genes were identified [15]. Based on a yeast library, 4695, 2641, and 2771 *Triticum aestivum* genes were screened that responded to freezing, salt, and osmotic stress, respectively, and *TaPR-1-1* was further identified as a key regulator in response to abiotic stress [13]. In *Tamarix hispida*, using a yeast expression system combined with high-throughput sequencing technology, 1224 potential genes were identified that conferred salt tolerance, and 21 were selected to verify their functions in *T. hispida* and *Arabidopsis thaliana* [16]. In summary, a yeast functional screening system can simply, quickly, and high-throughput screen candidate genes.

The aim of this study was to screen and characterize potential heat tolerance genes from potato using an *S. cerevisiae* heterologous expression system. With this system, we screened heat resistance genes simply and effectively. In addition, this study utilized PGSC data, qRT-PCR, and overexpression of candidate genes in yeast in response to high temperature for validation. The results showed that some potential potato heat-tolerant genes were also responsive to drought and salt stress. These genes may enhance multiple abiotic stress tolerance in potatoes. New insights are proposed to refine potato heat tolerance and other abiotic stresses.

## 2. Results

### 2.1. Determining S. tuberosum Heat-Responsive Genes Using a Yeast Functional Screening System

**Figure 1:**
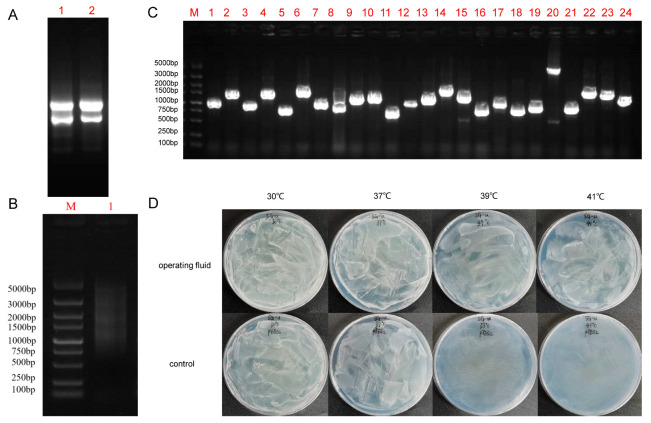
Potato cDNA library construction and high-temperature screening. (**A**) Total RNA was isolated from the whole potato plants, M: Maker; 1–2: Potato sample RNA; (**B**) Detection of synthesized cDNA quality, M: Maker; 1: Mixed purification results of three types of ds cDNA; (**C**) Detection of cDNA library inserts by PCR using pYES2-F and pYES2-R primers, M: Maker, 1–24: Randomly selected 24 clones for PCR detection; (**D**) Yeast libraries were treated at 37 °C, 39 °C, and 40 °C, and 39 °C was selected for treatment and sequencing.

Total RNA was isolated from potato seedling leaves treated with high temperature, and its quality was detected using agarose gel electrophoresis. The RNA was of high integrity with no degradation and therefore suitable for subsequent study (Figure 1A). This RNA was then reverse transcribed into cDNA for PCR amplification, and agarose gel electrophoresis was used to detect double stranded cDNA, which was successfully synthesized, with a length between 0.5 and 5 kb (Figure 1B). Then, we used homologous recombination to clone cDNA into the pYES2 vector plasmid for library construction. To test the library quality, 24 clones were randomly selected from the plates for colony PCR, and the results showed good library quality with library fragments’ average size of 1000 bp (Figure 1C). The library plasmid vector was transformed into *Saccharomyces cerevisiae* BY4741 to obtain a yeast working solution. To determine the optimal temperature for yeast high-temperature screening, we used a control temperature of 30 °C and high temperatures of 37 °C, 39 °C and 41 °C. The treated group still grew well at 39 °C, while the control group did not grow anymore (Figure 1D). Therefore, 39 °C was selected as the yeast library screening condition.

### 2.2. Gene Functional Annotation and Classification

**Figure 2:**
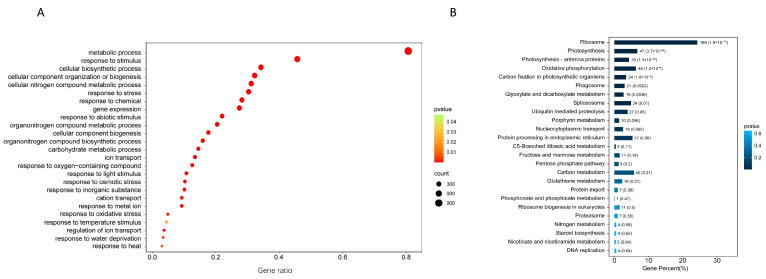
KEGG and GO analysis of genes responsive to high-temperature stress. (**A**) Biological processes of segregating genes; (**B**) KEGG pathway enrichment of isolated genes. Circle areas represent the relative numbers of isolated genes in the pathway; circle colors represent the range of Q values.

To screen candidate genes for potato response to high-temperature stress, we sequenced these amplicons on next-generation sequencing technology. After removing low quality and adaptor sequences, clean reads were obtained and immediately mapped to the potato reference genome. In total, 1602 (82.9%) known genes and 329 (17.1%) genes with unknown functions were detected in the expression library (Table S2). Genes encoding 34 heat shock factors (proteins), ribosomal proteins, 52 transcription factors (such as auxin response factor, basic-leucine zipper transcription factor family protein, zinc finger family protein), chaperone DNA J-domain superfamily protein, chlorophyll A/B binding protein, cytochrome P450, histone superfamily protein, lipid transfer proteins, 28 kinases, photosystem I subunit, and ubiquitin pathway proteins were annotated (Table S2). To further understand these genes’ functions, GO enrichment was performed. It was analyzed by GO enrichment, response to stimulus (GO: 0050896, *p* = 0.000398), cellular biosynthetic process (GO: 0044249, *p* = 0.000000), response to stress (GO: 0006950, *p* = 0.000017), response to abiotic stimulus (GO: 0009628, *p* = 0.000000), response to heat (GO: 0009408, *p* = 0.005846), and other pathways related to abiotic stress were enriched (Figure 2A). KEGG enrichment into nine pathways such as ribosome (ko03010, *p* = 1.91 × 10^−73^), photosynthesis (ko00195, *p* = 3.74 × 10^−26^), oxidative phosphorylation (ko00190, *p* = 1.23 × 10^−8^), carbon fixation in photosynthetic organisms (ko00710, *p* = 1.78 × 10^−6^), and ubiquitin-mediated proteolysis (ko04120, *p* = 4.96 × 10^−2^) were significantly enriched (Figure 2B). Additionally, we detected the MAPK signaling pathway (ko04016), phosphatidylinositol signaling system (ko04070), plant hormone signal transduction (ko04075), protein processing in endoplasmic reticulum (ko04141), and starch and sucrose metabolism (ko00500) (Figure 2B).

### 2.3. Gene Expression Analysis Based on PGSC Expression Data

**Figure 3:**
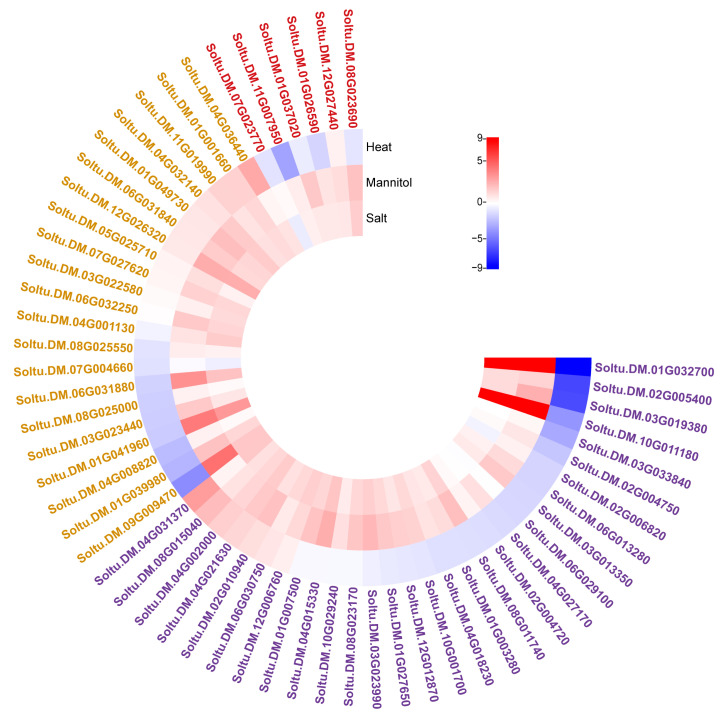
Expression analysis of genes based on PGSC expression data. Based on data from high temperature, drought, and salt stress in PGSC, Log2 fold change was used to indicate up- or down-regulated levels. The legend is log2 (FC), purple represents TF, yellow represents HSPs, and red represents kinases.

As we know, abiotic stresses, such as high temperature, high salt, and drought, often occur together. In the process of evolution, plants often adopt common regulatory factors or pathways to cope with multiple abiotic stresses simultaneously [17]. Totally, more than 51.48% (994/1931) DEGs were screened after heat, drought, or salt stress. Briefly, there were 541 (278 down-regulated and 263 up-regulated), 493 (203 down-regulated and 290 up-regulated), and 504 (101 down-regulated and 403 up-regulated) DEGs after heat, drought, and salt stress, respectively (Table S3). Additionally, 260 DEGs were commonly detected in all three stresses, and there were 266, 118, and 93 DEGs co-expressed in the salt and drought, heat and drought, and heat and salt groups, respectively. Further analysis revealed more than 95.8% (504/526) of DEGs (411 up-regulated and 93 down-regulated) were accordant in the salt and drought group while only 57.5% (203/353) and 47.1% (178/378) DEGs were accordantly regulated in heat and salt, and heat and drought, respectively. Among them, the expression levels of 30, 21, and 6 genes encoding TFs, HSP, and kinase, respectively, were significantly changed after heat, salt, or drought stress (Figure 3 and Table 1). In detail, 15 down- and 5 up-regulated TF genes were detected after heat stress, while there were 17 and 21 up-regulated and no down-regulated genes detected after salt and osmotic stress, respectively (Figure 3 and Table 1). Brassinosteroid and auxin play critical roles in heat stress response, and brassinosteroid signaling positive regulator (BZR1) family protein and auxin response factors (ARF) have been repeatedly proven to participate in high-temperature stress in many plants [18,19]. In this study, *Soltu.DM.02G006820* encoding BZR1 protein and two copies of ARF8 (*Soltu.DM.02G004750* and *Soltu.DM.02G004720*) declined 24 h after heat stress (Table 1). As previously reported, the DREB subfamily of ERF/AP2 transcription factor plays a pivotal role in plant abiotic stress regulation.

Two copies (*Soltu.DM.04G021630* and *Soltu.DM.08G015040*) encoding the DREB subfamily were significantly induced after heat, salt, and osmotic stresses (Table 1). Among the 21 changed HSP genes after treatments, all were induced after salt and osmotic stress except *Soltu.DM.07G004660*, which encoded the chaperonin 10 protein, while there were three up-regulated and nine down-regulated genes significantly induced after heat stress (Table 1). For kinase, *Soltu.DM.08G023690*, homologous of *SnRK2.6* and *Soltu.DM.12G027440*, encoding SOS3-interacting protein 3, were significantly induced after salt or osmotic stress, while the expression levels of *Soltu.DM.11G007950* encoding a transmembrane kinase 1 and two genes (*Soltu.DM.07G023770* and *Soltu.DM.01G026590*) encoding protein kinase superfamily proteins declined after heat stress (Table 1).

### 2.4. Expression Pattern of Selected Potato Genes under Heat, Salt, and Drought Stresses

**Figure 4:**
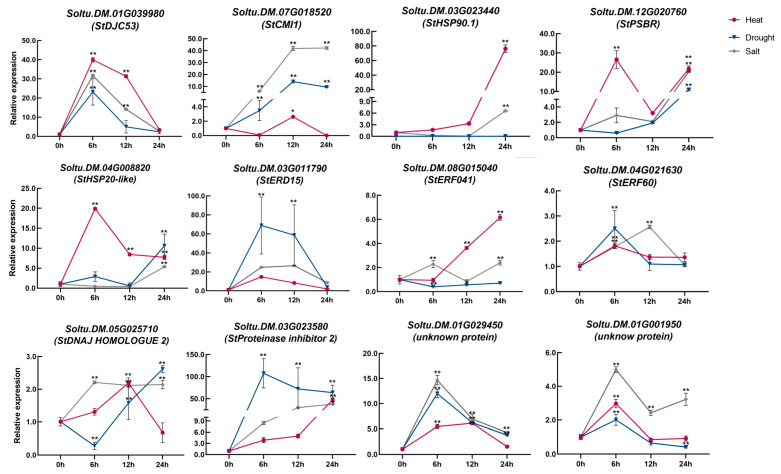
Expression patterns of candidate genes for heat resistance in potato subjected to different stress treatments. Two-week-old potato seedlings were treated with 35 °C, 120 mM NaCl, and 20% PEG 6000, and samples were taken at 0 h, 6 h, 12 h, and 24 h. *StEF1α* was used as a control with three independent biological replicates. Red, blue, and gray represent heat stress, drought stress, and salt stress, respectively. *: *p* < 0.05; **: *p* < 0.01.

To further explore candidate genes’ expression patterns under high temperature, drought, and salt stress, 12 genes were randomly selected and validated by qRT-PCR. Expression of the 12 selected genes was induced by high-temperature stress, but the timing of the response to high temperature differed (Figure 4). Most of these genes have a tendency to rise and then fall in response to high temperature induction, such as *Soltu.DM.01G039980* (*Chaperone DnaJ-domain superfamily protein 53, DJC53*), *Soltu.DM.03G011790* (*Early Responsive to Dehydration 15, ERD15*), *Soltu.DM.01G029450* (*Hypothetical proteins*), and *Soltu.DM.01G001950* (*Hypothetical proteins*). Additionally, some genes were only induced at separate times in the early or late stage, for example, *Soltu.DM.03G023440* (*Heat shock protein 90.1, HSP90.1*) and *Soltu.DM.03G023580* (*Proteinase inhibitor 2*) were more strongly responsive at 24 h, while *Soltu.DM.07G018520* (*Kunitz trypsin inhibitor 5, KTI5*) and *Soltu.DM.12G020760* (*PHOTOSYSTEM II SUBUNIT R, PSBR*) were induced more strongly at 6 h and 12 h, respectively. We suggest that this might be due to different genes’ functions being exerted at different times during heat stress (Figure 4).

Meanwhile, we also detected these genes in response to drought or salt stress using qRT-PCR, with the same expression pattern of *Soltu.DM.04G021630* (*ERF60*) in response to high temperature and drought stress. Moreover, *DJC53*, *ERD15*, and two *hypothetical proteins* showed identical expression patterns under the three stresses (Figure 4). High temperatures are often accompanied by drought, and the same regulatory mechanisms may exist; therefore, we speculated that the same mechanism maybe operating during high temperature, drought, and salt stress, but this needs to be further explored.

### 2.5. High-Temperature Candidate Genes’ Transfer Enhances Heat Tolerance in Saccharomyces cerevisiae

**Figure 5:**
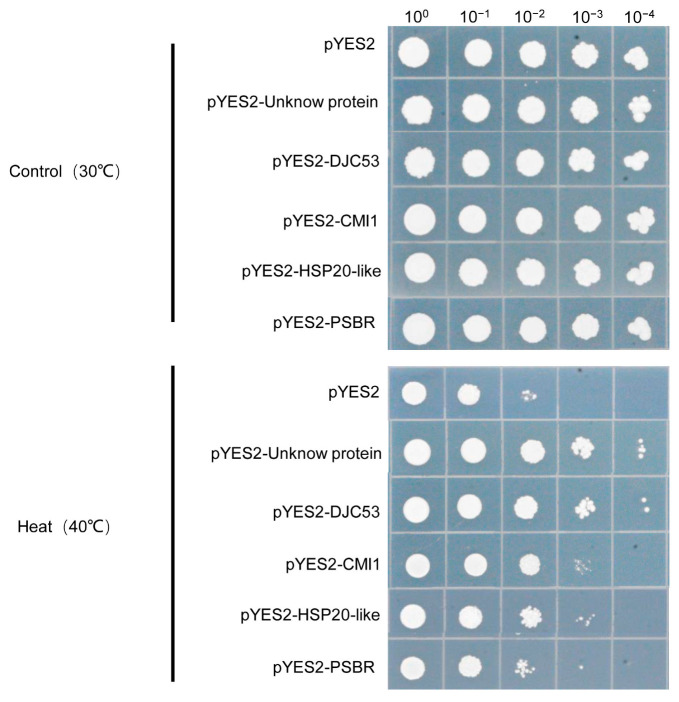
Transfer high-temperature candidate genes enhances heat tolerance in *S. cerevisiae*. The empty pYES vector and the vector with the candidate genes were transformed into BY4741, diluted, and cultured at 30 °C (control) and 40 °C (heat stress) for 3 d.

To further verify the accuracy of the screened high-temperature candidate genes, five genes were randomly selected for high-temperature stress validation in *Saccharomyces cerevisiae*. They were cloned into the pYES2 vector and transformed into *S. cerevisiae* BY4741 for high-temperature stress. There were no significant differences in growth among the five transgenic *S. cerevisiae* under normal growth conditions compared with the empty vector, while *S. cerevisiae* with over-expression of *DJC53*, *HSP20-like*, *PSBR*, *CMI1*, and *hypothetical protein* genes had a significantly higher survival rate compared to *S. cerevisiae* with the empty vector under 40 °C (Figure 5). In summary, the five genes significantly enhanced *S. cerevisiae*, proving that the screened genes were effective.

## 3. Discussion

To screen candidate genes responding to abiotic stresses such as heat and drought, many approaches such as QTL mapping and transcriptome sequencing have been used in various crops [20,21]. Potatoes, as homologous tetraploids, have a complex genome and high heterozygosity, resulting in poor candidate genes screening through QTL and transcriptome sequencing. Fortunately, many examples have been recently reported utilizing yeast expression systems in plants that can quickly and efficiently screen stress-responsive genes for high-throughput screening [10,11,12]. We obtained 1931 that genes responded to high temperature using the yeast functional screening system in potato (Table S2). GO enrichment indicated that these responsive genes were mainly enriched in metabolic process, response to stimulus, in oxidative stress, and response to high temperature (Figure 2B). In a previous study, only 95 heat-resistant candidate genes were identified by high-throughput sequencing of a yeast library constructed after high-temperature stress [15]. Compared to that study, we obtained more genes that respond to high temperatures (1931), including some that had been screened previously, such as heat shock proteins, ion-associated genes, and photosynthesis-related genes (Table S2).

Plants share common regulatory signals or pathways in response to abiotic stresses such as high temperature, drought, and high salt [22,23]. Among the 1931 high temperature responsive genes we screened, more than half responded to both drought and high salt stress simultaneously (Table S3). To further verify the accuracy of these data, we randomly selected 12 candidate genes for qRT-PCR validation and showed that these genes responded to at least one abiotic stress (Figure 4). Among these 12 genes, *HSP90.1* was significantly induced by heat (70-fold) and salt stress (7-fold), while it was not significantly induced by drought stress (Figure 4). In a previous study, an HSP90.1 promoter containing a cis-acting element of HvSHN1 improved heat, salt, and drought tolerance in tobacco [24]. In *Arabidopsis*, it was also found that the interaction between HSP90.1 and ROF1 (FKBP62) could affect HSFA2 expression to enhance heat tolerance [25]. Additionally, HSP90.1 enhanced salt tolerance in *S. cerevisiae*, and *Arabidopsis* HSP90 was engaged in salt tolerance by HOP1 and HOP2, which affected its nucleoplasmic distribution [26,27]. *ERD15* was simultaneously induced by high temperature, drought, and high salt stress with similar patterns (Figure 4), which suggests that ERF15 is the core regulator of factors regulating abiotic stresses. ERD15 was found to be a negative ABA signaling regulator in *Arabidopsis*, affecting stomatal movement and drought resistance [28,29]. In soybean, *GmERD15B* overexpression enhanced salt tolerance by increasing the expression levels of genes related to ABA-signaling, proline content, and cation transport [30]. *ERF60* and *ERF041*, AP2/ERF family TF, were induced by high temperatures, with the highest induction multiplicity of 6-fold and 2-fold, respectively (Figure 4). ERF60 has an important role in response to temperature stress in pea using RNA-seq [31]. Moreover, *ERF60* overexpression enhances drought and salt tolerance in Arabidopsis seedlings [32].

To further confirm candidate genes’ roles in high-temperature responsiveness, we randomly selected five genes for heterologous expression in yeast and found that all these genes enhanced high-temperature tolerance of yeast to some extent (Figure 5). DJC53, a Chaperone DnaJ domain superfamily protein, is involved in regulating the response of plant cells’ responses to heat stress and negatively regulates heat tolerance in *Arabidopsis* [33]. This is contrary to our yeast results, possibly due to different roles in different species. CMI1, a Ca^2+^ binding protein, mediates auxin responses during plant growth and was significantly upregulated in over-expression lines of AtMBF1c, which is a positive regulatory factor for heat stress [34], suggesting that AtCMI1 may be involved in high-temperature regulation. Additionally, *AtCMI1* may serve as a downstream gene of AtMYB60 and AtZAT12, regulating abiotic stress processes such as drought and osmotic stress [35,36]. These results highlight that research on abiotic stress in CMI1 has been limited to indirect evidence. Our results showed that this gene significantly enhanced the *S. cerevisiae* survival rate under high temperatures, proving that CMI1 responded to and enhanced *S. cerevisiae* heat tolerance. PSBR is a PHOTOSYSTEM II SUBUNIT R subunit involved in PS II assembly, which contains multiple components [37]. The qRT-PCR results showed that the expression level of this gene was up-regulated about 25-fold after 6 h of high-temperature treatment, which significantly improved its heat tolerance after transfection into *S. cerevisiae*. In addition, the gene was also induced by drought and salt stress. Previous studies have shown that the external components of PS II play an important role in responding to abiotic stress [38]. In our study, the expression level of *HSP20-like* was significantly induced by high temperature, up to 20-fold, and was transfected into *S. cerevisiae* to enhance its heat tolerance. HSP20-like has been shown to enhance heat tolerance in *Arabidopsis thaliana* [39], and *OsHSP20* overexpression improved heat and salt tolerance in rice [40].

In summary, our 12 selected genes may be involved in abiotic stress regulation in potato and will be the focus of subsequent studies. This study is important for screening potato stress-responsive functional genes and provides new insights for improving the potato abiotic stress regulatory network.

## 4. Materials and Methods

### 4.1. Materials, Stress Treatments, and RNA Isolation

Nodal explants of potato plants (*S. tuberosum* cv. Eshu #3) were grown in Murashige and Skoog (MS) medium, containing 3% sucrose and 0.7% agar for two weeks, and then plants were carefully separated from the solid agar media and transferred to a liquid ½MS medium containing 0.5% sucrose (L½MS) for two days. All plants were then transferred to pots containing soil and placed in a light incubator with relative humidity of 50–60%, temperature of 22 °C/18 °C, and a 16 h photoperiod for 25 days. Finally, we changed the temperature to 35 °C/28 °C for three days. Then we collected 10 mature leaves from each of 10 plants, placed them in liquid nitrogen for 30 min, and stored them at −80 °C until further use [41]. Total RNA was isolated from the samples collected before with Trizol (Invitrogen, Thermo Fisher Scientific Inc., Waltham, MA, USA). The total RNA’s integrity was detected using 1% agarose gel electrophoresis, and its concentration was estimated by using nanodrop (Thermo Scientific).

### 4.2. Yeast cDNA Expression Library Construction

Total RNA was extracted using Trizol according to the manufacturer’s instructions. To construct the cDNA library, the total RNA of all samples was reverse transcribed into cDNA using a SMART™ cDNA Library Construction Kit, and then the cDNA was further PCR amplified using the primers P1-F, P2-F, P3-F, and P4-R (Table S1). The BY4741 vector used in this study was cut using the restriction enzyme *Hind* III and *Xba* I and then rebuilt with the purified PCR product. Successful transformants were selected as cDNA library screened on Luria–Bertani (LB) agar plates supplemented with 100 µg/mL ampicillin. The constructed cDNA library was then converted into the yeast strain BY4741 using a Yeastmaker™ Yeast Transformation System 2 kit (Clontech, Mountain View, CA, USA) according the Yeastmaker™ Yeast Transformation System instructions. The transformed yeast was inoculated on SD-Ura defective solid medium with glucose as the carbon source, and cultured upside down at 30 °C for 72–100 h to prepare the working solution, which is the yeast cDNA library. The library quality was checked using a Yeast Colony Rapid Detection Kit (Nanjing Ruiyuan Biotechnology Co., Ltd., Nanjing, China), following its instructions. To determine the screening temperature, the SD-Ura plates inoculated with the yeast working solution and control strains (transformed with empty body) were placed in incubators at 30 °C, 37 °C, 39 °C, and 41 °C, and the screening temperature was determined based on their observed growth conditions after 3 days. Yeast colonies were collected by washing with YPDA solution (YPD + 25% glycerol) after high-temperature screening and then the pYES2 plasmid was isolated, which acted as the amplification template using primers P1 and P4. Then, these PCR products were used for Illumina high-throughput sequencing on Illumina HiSeq™ 2500 (Biomarker Technologies Corporation, Beijing, China).

### 4.3. DNA Sequencing and Gene Annotation

Clean data were obtained after the removal of vectors and adaptor sequences using VecScreen (http://www.ncbi.nlm.nih.gov/VecScreen/VecScreen.html) and mapping to the potato reference genome database (http://spuddb.uga.edu/dm_v6_1_download.shtml). The rest sequences were then used to predict possible open reading frames (ORF) using GENSCAN (http://genes.mit.edu/GENSCAN.html). Additionally, the non-redundant sequence database at NCBI (http://www.ncbi.nlm.nih.gov/blast/) was used to assign gene function by performing homology searches. All these annotated sequences were further mapped to GO categories, and BlaST2GO was used to assign KEGG pathways. Furthermore, genes’ encoding transcription factors, kinases, and heat shock proteins were manually screened according their annotation.

To further analyze the expression of these genes, expression levels were investigated based on PGSC expression data (NCBI accession: SRA030516). For heat, salt, and osmotic stress, SRR122112 (control for heat), SRR122115 (35 °C, 24 h), SRR122131 (control for salt and osmotic), SRR122120 (150 mM NaCl, 24 h), and SRR122128 (260 μM mannitol, 24 h) were selected. To analyze the effect of stress treatment on gene expression levels, the differentially expressed genes (DEGs) were screened using the following parameters: absolute value of log2 (fold change) > 1 and the TPM > 10 in at least one sample between control and treatments.

### 4.4. qRT-PCR Analysis

To detect whether screened genes responded to high temperature, the expression levels of 20 randomly selected genes before and after high temperature were detected by qRT-PCR. Briefly, 1 μg of total RNA (isolated prior) was used for cDNA synthesis using a Transcriptor First-Strand cDNA Synthesis Kit (Roche, Basel, Switzerland) [42]. The qRT-PCR was performed as described previously. *StEF1α* and *StGAPDH* were used as internal controls, and the relative expression levels were calculated using the 2^−ΔΔCt^ method [42,43]. The gene-specific primers used in this study are shown in Table S1. Three biological replications with three technical replicates were performed on each reaction.

### 4.5. High-Temperature Sensitivity Assays in Yeast Cells

To test whether these screened genes can enhance high-temperature tolerance of yeast, we randomly selected seven genes and transformed them into yeast. The yeast transformants were precultured for 24 h on SD-Ura liquid medium supplemented with glucose at 30 °C and then transferred onto SD-Ura liquid medium supplemented with galactose with vigorous shaking for 36 h at 30 °C to reach a density of 1.0 at OD600. These cells were serially diluted in 10-fold steps and 1 µL aliquots of each were finally spotted onto SD-Ura agar medium at normal temperature (30 °C) and high temperature (40 °C) for 2 to 5 days.

## 5. Conclusions

We screened potato genes for high-temperature tolerance by constructing potato cDNA yeast libraries for high-temperature stress. A total of 1931 high-temperature candidate genes were screened. The results of KEGG and GO enrichment showed that these genes were enriched in pathways related to stimulation. According to PGSC data, some differential genes are responsive to high temperature, drought, and salt stress. Twelve of these genes were validated by qRT-PCR, and the results showed that they were all induced by high temperature and other abiotic stresses. Finally, five of these genes were transformed into *S. cerevisiae*, and the results showed that the expression of these genes enhanced the heat tolerance of *S. cerevisiae* under high-temperature stress, preliminarily exploring the functions of these genes. This study provides new clues for analyzing the potato’s heat tolerance mechanism, which is of great significance for potato heat tolerance breeding.

## Figures and Tables

**Table 1:** DEGs encoding TFs, HSP, and Kinase after heat, salt, and osmotic stress at 24 h based on PGSC expression data. CK ① is control for salt stress and mannitol, CK ② is control for heat stress.

Transcript ID	FPKM					Log2 (Fold Change)			Homologous of *Arabidopisis*	FunctionalAnnotation	Type
	CK ①	Salt	Mannitol	CK ②	Heat	Salt	Osmotic	Heat			
*Soltu.DM.01G032700*	0.01	10.42	6.82	7.39	0.01	10.03	9.41	−9.53	*AT2G45660.1*	AGAMOUS-like 20	TF
*Soltu.DM.02G005400*	4.24	9.25	11.38	24.45	0.28	1.13	1.42	−6.47	*AT2G17040.1*	NAC domain containing protein 36	TF
*Soltu.DM.03G019380*	7.30	16.39	43.31	17.69	0.21	1.17	2.57	−6.36	*AT1G14440.2*	homeobox protein 31	TF
*Soltu.DM.10G011180*	0.01	6.84	11.78	6.53	0.47	9.42	10.20	−3.78	*AT1G58110.2*	Basic-leucine zipper (bZIP) transcription factor family protein	TF
*Soltu.DM.03G033840*	12.54	12.52	15.35	27.29	3.13	0.00	0.29	−3.12	*AT1G73830.1*	BR enhanced expression 3	TF
*Soltu.DM.02G004750*	4.18	4.70	7.29	11.18	2.69	0.17	0.80	−2.06	*AT5G37020.1*	auxin response factor 8	TF
*Soltu.DM.02G006820*	74.04	54.67	102.37	224.76	75.59	−0.44	0.47	−1.57	*AT1G75080.2*	Brassinosteroid signaling positive regulator (BZR1) family protein	TF
*Soltu.DM.06G013280*	9.46	13.22	21.68	51.89	17.67	0.48	1.20	−1.55	*AT5G52510.1*	SCARECROW-like 8	TF
*Soltu.DM.03G013350*	18.07	29.59	60.32	13.38	4.80	0.71	1.74	−1.48	*AT3G56400.1*	WRKY DNA-binding protein 70	TF
*Soltu.DM.06G029100*	98.07	90.66	93.74	123.78	45.82	−0.11	−0.07	−1.43	*AT3G20770.1*	Ethylene insensitive 3 family protein	TF
*Soltu.DM.04G027170*	6.33	6.59	13.10	19.29	7.55	0.06	1.05	−1.35	*AT5G44080.1*	Basic-leucine zipper (bZIP) transcription factor family protein	TF
*Soltu.DM.02G004720*	9.66	11.29	12.69	11.94	5.07	0.23	0.39	−1.24	*AT5G37020.1*	auxin response factor 8	TF
*Soltu.DM.08G011740*	17.52	46.81	72.52	126.37	54.59	1.42	2.05	−1.21	*AT5G11270.1*	overexpressor of cationic peroxidase 3	TF
*Soltu.DM.01G003280*	9.34	15.34	20.53	11.14	5.00	0.72	1.14	−1.16	*AT2G02080.1*	indeterminate (ID)-domain 4	TF
*Soltu.DM.04G018230*	12.95	23.50	24.89	60.41	27.20	0.86	0.94	−1.15	*AT3G52250.1*	Duplicated homeodomain-like superfamily protein	TF
*Soltu.DM.10G001700*	13.12	31.28	35.32	34.66	17.76	1.25	1.43	−0.96	*AT5G54680.1*	basic helix-loop-helix (bHLH) DNA-binding superfamily protein	TF
*Soltu.DM.12G012870*	7.11	12.35	20.92	29.44	15.99	0.80	1.56	−0.88	*AT5G23090.4*	nuclear factor Y, subunit B13	TF
*Soltu.DM.01G027650*	23.27	55.46	79.25	207.26	121.61	1.25	1.77	−0.77	*AT3G08505.1*	zinc finger (CCCH-type/C3HC4-type RING finger) family protein	TF
*Soltu.DM.03G023990*	2.14	6.24	10.47	14.83	9.96	1.55	2.29	−0.57	*AT4G32730.2*	Homeodomain-like protein	TF
*Soltu.DM.08G023170*	7.11	17.31	25.29	19.21	15.91	1.28	1.83	−0.27	*AT5G45420.1*	Duplicated homeodomain-like superfamily protein	TF
*Soltu.DM.10G029240*	25.55	40.36	51.67	27.52	22.88	0.66	1.02	−0.27	*AT5G06770.1*	KH domain-containing protein/zinc finger (CCCH type) family protein	TF
*Soltu.DM.04G015330*	5.38	19.64	32.32	59.65	50.36	1.87	2.59	−0.24	*AT2G33550.1*	Homeodomain-like superfamily protein	TF
*Soltu.DM.01G007500*	35.41	88.80	131.85	183.06	154.98	1.33	1.90	−0.24	*AT5G13180.1*	NAC domain containing protein 83	TF
*Soltu.DM.12G006760*	5.54	16.15	13.16	20.90	30.15	1.54	1.25	0.53	*AT5G65070.1*	K-box region and MADS-box transcription factor family protein	TF
*Soltu.DM.06G030750*	7.58	18.00	12.37	6.11	11.06	1.25	0.71	0.86	*AT2G28510.1*	Dof-type zinc finger DNA-binding family protein	TF
*Soltu.DM.02G010940*	8.55	16.60	34.00	19.18	39.98	0.96	1.99	1.06	*AT3G27010.1*	TEOSINTE BRANCHED 1, cycloidea, PCF (TCP)-domain family protein 20	TF
*Soltu.DM.04G021630*	96.79	304.78	316.91	477.46	1196.88	1.65	1.71	1.33	*AT4G39780.1*	Integrase-type DNA-binding superfamily protein	TF
*Soltu.DM.04G002000*	6.21	15.14	15.82	7.72	23.04	1.29	1.35	1.58	*AT1G25440.1*	B-box type zinc finger protein with CCT domain	TF
*Soltu.DM.08G015040*	5.29	10.68	9.98	3.50	15.45	1.01	0.92	2.14	*AT5G11590.1*	Integrase-type DNA-binding superfamily protein	TF
*Soltu.DM.04G031370*	0.79	2.68	1.07	4.67	40.22	1.76	0.42	3.11	*AT2G14210.1*	AGAMOUS-like 44	TF
*Soltu.DM.09G009470*	1.05	3.60	23.70	46.41	2.74	1.78	4.50	−4.08	*AT1G53540.1*	HSP20-like chaperones superfamily protein	HSP
*Soltu.DM.01G039980*	30.11	73.87	116.80	80.48	12.47	1.29	1.96	−2.69	*AT1G56300.1*	Chaperone DnaJ-domain superfamily protein	HSP
*Soltu.DM.04G008820*	31.69	36.28	42.81	195.65	36.36	0.20	0.43	−2.43	*AT5G37670.1*	HSP20-like chaperones superfamily protein	HSP
*Soltu.DM.01G041960*	0.56	5.19	10.14	13.27	3.63	3.22	4.19	−1.87	*AT4G10250.1*	HSP20-like chaperones superfamily protein	HSP
*Soltu.DM.03G023440*	11.95	21.31	39.52	117.25	33.65	0.83	1.73	−1.80	*AT5G52640.1*	heat shock protein 90.1	HSP
*Soltu.DM.08G025000*	12.16	14.04	16.68	18.35	5.47	0.21	0.46	−1.75	*AT5G47590.1*	Heat shock protein HSP20/alpha crystallin family	HSP
*Soltu.DM.06G031880*	1.28	4.90	14.30	14.85	4.87	1.93	3.48	−1.61	*AT1G07400.1*	HSP20-like chaperones superfamily protein	HSP
*Soltu.DM.07G004660*	145.00	98.96	129.47	114.99	52.36	−0.55	−0.16	−1.13	*AT1G14980.1*	chaperonin 10	HSP
*Soltu.DM.08G025550*	65.78	89.42	99.43	154.44	76.63	0.44	0.60	−1.01	*AT4G22670.1*	HSP70-interacting protein 1	HSP
*Soltu.DM.04G001130*	5.38	16.50	10.40	19.10	14.06	1.62	0.95	−0.44	*AT3G08910.1*	DNAJ heat shock family protein	HSP
*Soltu.DM.06G032250*	20.15	51.01	66.49	45.28	48.49	1.34	1.72	0.10	*AT5G58740.1*	HSP20-like chaperones superfamily protein	HSP
*Soltu.DM.03G022580*	23.21	53.31	34.51	51.15	57.33	1.20	0.57	0.16	*AT5G53400.1*	HSP20-like chaperones superfamily protein	HSP
*Soltu.DM.07G027620*	914.03	2243.58	2560.85	4803.50	5901.53	1.30	1.49	0.30	*AT5G56000.1*	HEAT SHOCK PROTEIN 81.4	HSP
*Soltu.DM.05G025710*	1122.25	1514.19	2365.67	3477.91	4490.32	0.43	1.08	0.37	*AT5G22060.1*	DNAJ homologue 2	HSP
*Soltu.DM.12G026320*	5.16	31.78	32.64	31.73	53.42	2.62	2.66	0.75	*AT1G53540.1*	HSP20-like chaperones superfamily protein	HSP
*Soltu.DM.06G031840*	25.66	61.71	80.93	122.40	212.18	1.27	1.66	0.79	*AT1G07400.1*	HSP20-like chaperones superfamily protein	HSP
*Soltu.DM.01G049730*	7.02	17.61	29.55	12.56	22.20	1.33	2.07	0.82	*AT4G39150.2*	DNAJ heat shock N-terminal domain-containing protein	HSP
*Soltu.DM.04G032140*	4.12	13.02	13.34	10.61	20.67	1.66	1.69	0.96	*AT4G07990.1*	Chaperone DnaJ-domain superfamily protein	HSP
*Soltu.DM.11G019990*	22.76	45.21	43.24	9.09	24.46	0.99	0.93	1.43	*AT5G19855.1*	Chaperonin-like RbcX protein	HSP
*Soltu.DM.01G001660*	58.79	130.16	142.28	49.45	137.02	1.15	1.28	1.47	*AT2G34860.2*	DnaJ/Hsp40 cysteine-rich domain superfamily protein	HSP
*Soltu.DM.04G036440*	64.68	122.78	83.19	11.34	77.58	0.92	0.36	2.77	*AT1G75690.1*	DnaJ/Hsp40 cysteine-rich domain superfamily protein	HSP
*Soltu.DM.07G023770*	21.39	13.16	24.89	25.42	12.33	−0.70	0.22	−1.04	*AT1G16670.1*	Protein kinase superfamily protein	Kinase
*Soltu.DM.11G007950*	13.36	19.69	20.75	39.74	4.01	0.56	0.63	−3.31	*AT1G66150.1*	transmembrane kinase 1	Kinase
*Soltu.DM.01G037020*	21.50	35.83	72.27	25.57	15.55	0.74	1.75	−0.72	*AT5G58140.1*	phototropin 2	Kinase
*Soltu.DM.01G026590*	14.58	24.37	26.87	15.18	5.15	0.74	0.88	−1.56	*AT3G51990.1*	Protein kinase superfamily protein	Kinase
*Soltu.DM.12G027440*	59.20	101.21	125.91	23.31	31.84	0.77	1.09	0.45	*AT4G30960.1*	SOS3-interacting protein 3	Kinase
*Soltu.DM.08G023690*	11.93	35.87	45.58	47.01	24.58	1.59	1.93	−0.94	*AT4G33950.1*	Protein kinase superfamily protein	Kinase

## Data Availability

All of the data generated or analyzed during this study are included in this published article. Nucleotide sequence data reported are available in the NCBI databases under the accession number [PRJNA1023083].

## Supplementary Materials

The following supporting information can be downloaded at: https://www.mdpi.com/article/10.3390/plants12213712/s1, Table S1: All primers used in this study; Table S2: Information about all candidate genes; Table S3: DEGs screened after heat, drought, and salt stress.
